# Regulation of Na_v_1.7: A Conserved *SCN9A* Natural Antisense Transcript Expressed in Dorsal Root Ganglia

**DOI:** 10.1371/journal.pone.0128830

**Published:** 2015-06-02

**Authors:** Jennifer Koenig, Robert Werdehausen, John E. Linley, Abdella M. Habib, Jeffrey Vernon, Stephane Lolignier, Niels Eijkelkamp, Jing Zhao, Andrei L. Okorokov, C. Geoffrey Woods, John N. Wood, James J. Cox

**Affiliations:** 1 Molecular Nociception Group, Wolfson Institute for Biomedical Research, University College London, Gower Street, London, United Kingdom; 2 Department of Anesthesiology, Medical Faculty, Heinrich-Heine-University Düsseldorf, Moorenstr. 5, Düsseldorf, Germany; 3 Laboratory for Translational Immunology, UMC Utrecht, Utrecht, The Netherlands; 4 Wolfson Institute for Biomedical Research, University College London, Gower street, London, United Kingdom; 5 Cambridge Institute for Medical Research, University of Cambridge, Hills Road, Cambridge, United Kingdom; 6 Department of Molecular Medicine and Biopharmaceutical Sciences, Graduate School of Convergence Science and Technology, College of Medicine, Seoul National University, Seoul, South Korea; The Hebrew University Medical School, ISRAEL

## Abstract

The Na_v_1.7 voltage-gated sodium channel, encoded by *SCN9A*, is critical for human pain perception yet the transcriptional and post-transcriptional mechanisms that regulate this gene are still incompletely understood. Here, we describe a novel natural antisense transcript (NAT) for *SCN9A* that is conserved in humans and mice. The NAT has a similar tissue expression pattern to the sense gene and is alternatively spliced within dorsal root ganglia. The human and mouse NATs exist in cis with the sense gene in a tail-to-tail orientation and both share sequences that are complementary to the terminal exon of *SCN9A*/*Scn9a*. Overexpression analyses of the human NAT in human embryonic kidney (HEK293A) and human neuroblastoma (SH-SY5Y) cell lines show that it can function to downregulate Na_v_1.7 mRNA, protein levels and currents. The NAT may play an important role in regulating human pain thresholds and is a potential candidate gene for individuals with chronic pain disorders that map to the *SCN9A* locus, such as Inherited Primary Erythromelalgia, Paroxysmal Extreme Pain Disorder and Painful Small Fibre Neuropathy, but who do not contain mutations in the sense gene. Our results strongly suggest the *SCN9A* NAT as a prime candidate for new therapies based upon augmentation of existing antisense RNAs in the treatment of chronic pain conditions in man.

## Introduction

Following the cataloguing of the human genome and transcriptome it has become apparent that there are probably more genes in the human genome that encode regulatory RNAs than those that encode proteins [1]. One major class of regulatory RNA genes contains the long non-coding RNAs (lncRNAs), of which natural antisense transcripts (NATs) are an important subset. NATs can be defined as processed transcripts that are complementary to the corresponding processed sense transcript in exonic regions [2]. NATs can exist in cis or trans to the target gene and are relatively common, with approximately 70% of all genomic loci showing evidence of transcription from both sense and antisense strands [3]. Prominent examples of NATs include *Tsix* (the NAT for *Xist*), *Wrap53* (the NAT for p53) and *BACE1-AS* (the NAT for beta-secretase-1) [4–6]. In the pain field, a NAT was recently reported for the voltage-dependent potassium channel Kcna2 [7]. This NAT is expressed in rat dorsal root ganglion (DRG) neurons and is upregulated in response to peripheral nerve injury. The increase in NAT levels downregulates Kcna2, attenuating total voltage-gated potassium currents, increasing excitability in DRG neurons and producing neuropathic pain symptoms.

We were interested to discover whether a NAT exists for *SCN9A*, another pain-related gene, which encodes the Na_v_1.7 voltage-gated sodium channel. Previously we reported that recessive loss of function mutations in this channel result in a complete inability to perceive pain (CIP) [8]. In addition to being pain-free from birth, *SCN9A*-CIP patients also lack a sense of smell, but are otherwise normal [9]. Consequently, this channel has been identified as a promising target in the pharmaceutical industry for the development of new analgesic drugs [10]. In contrast to the pain-free phenotype, there are also debilitating painful Mendelian disorders resulting from gain of function of Na_v_1.7, such as Inherited Primary Erythromelalgia (IEM), Paroxysmal Extreme Pain Disorder (PEPD) and painful small fibre neuropathy [11–13]. We considered that if a NAT did exist for *SCN9A*, then perhaps it played a role in regulating Na_v_1.7 protein levels and hence altering responses to painful stimuli.

In this study, using an *in silico* approach to inform the design of RT-PCR reactions, we have cloned a NAT for *SCN9A* that is conserved in humans and mice. The tissue expression profile of the NAT is similar to the sense gene, indicating that it may play an important functional role. Overexpression analyses of the NAT have shown that it reduces Na_v_1.7 mRNA, protein and currents. This NAT is therefore a potentially interesting candidate gene for IEM, PEPD and small fibre neuropathy patients that lack pathogenic mutations in *SCN9A* [14,15].

## Results

### Cloning the *SCN9A*/*Scn9a* natural antisense transcripts

**Fig 1:**
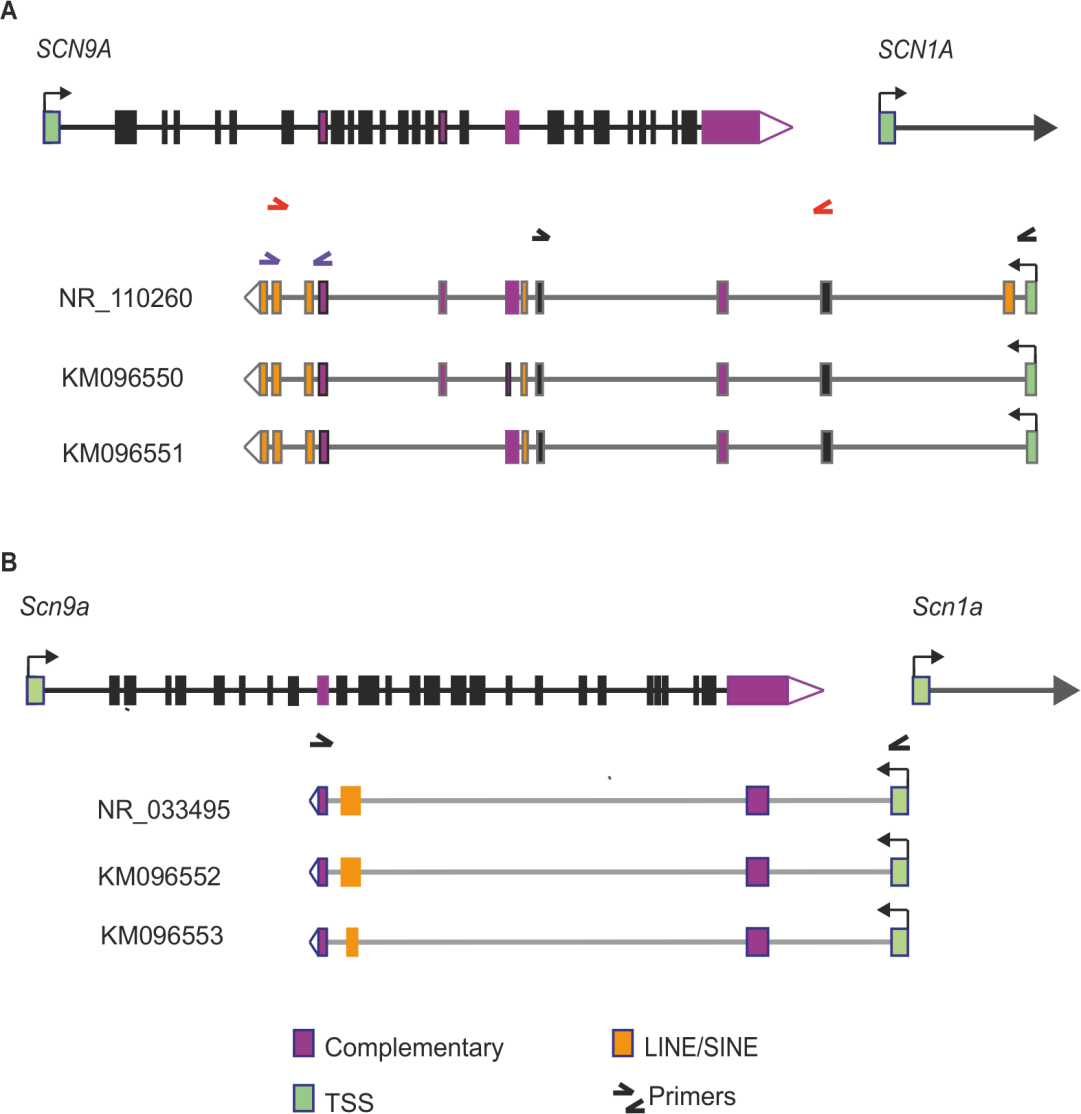
Genomic organization of the human (A) and mouse (B) *SCN9A*/*Scn9a* natural antisense transcripts (NATs). The sense genes are shown at the top of each panel with the NAT splice variants shown below. The sense genes and NATs are arranged in a tail-to-tail orientation (i.e. 3’ ends overlapping). Transcriptional start sites (TSS) and direction of transcription are denoted by green exons and arrows respectively; overlapping regions between sense and NAT sequences are shown by purple exons; LINE and SINE repeat sequences are shown as orange exons; and the primer pairs used to amplify the respective NAT sequences are also highlighted.

*In silico* analyses of the human and mouse *SCN9A*/*Scn9a* gene footprints using the UCSC genome browser identified several expressed sequence tags (ESTs) that were partially complementary to exonic regions of the sense gene. Alignment of the longest human EST, BC051759, to the genomic sequence indicated a cDNA comprised of 12 exons; four of which were complementary to and partly or wholly overlapped exons from *SCN9A* (S1 Fig); and with five exons containing SINE and/or LINE repeat sequences. Exons were flanked with the canonical AG-GT splice acceptor and donor sites and the final exon contained an AAUAAA polyadenylation signal. In Genbank the assembly of ESTs has subsequently been annotated as LOC101929680 (NR_110260), which spans 220 kb on chromosome 2 and encodes an uncharacterized long non-coding RNA of 2305 bp (Fig 1A). Using human dorsal root ganglion cDNA as template we amplified two alternative splice variants, which were submitted to Genbank. Compared to NR_110260, the first splice variant (KM096550) excludes exon 2 and uses an alternative splice acceptor site within exon 7. The second splice variant (KM096551) excludes both exon 2 and exon 8 (Fig 1A). Interestingly, some *SCN9A* point mutations previously shown to cause the human monogenic pain disorders CIP, IEM and PEPD also change the sequence of the NAT (S1 Fig).

Analysis of the mouse genome also led to the identification of several ESTs that were antisense to *Scn9a*. For example, EST AK138532 indicated a cDNA comprised of four exons, one of which splices into a LINE repeat and two of which overlap *Scn9a* sense gene exons (NR_033495; Fig 1B). Similar to the human *SCN9A* NAT, exons were flanked with canonical AG-GT splice acceptor and donor sites and the final exon contained an AAUAAA polyadenylation signal. Both the human and mouse NATs contain sequences that overlap the final sense *SCN9A*/*Scn9a* exon, potentially indicating a conserved regulatory function of these NATs in man and mouse. Using mouse dorsal root ganglion cDNA as template we amplified the identical sequence to NR_033495 (KM096552, Fig 1B) as well as a splice variant (KM096553, Fig 1B), which uses an alternative splice donor site in exon 3.

*In silico* translation of the human and mouse NAT sequences shows that the longest potential open reading frames are 67 and 114 amino acids respectively (S2 Fig). The lack of a long open reading frame and the poor codon conservation is consistent with the definition of a long non-coding RNA [1].

### *Scn9a* sense and NAT genes have a similar tissue expression profile

**Fig 2:**
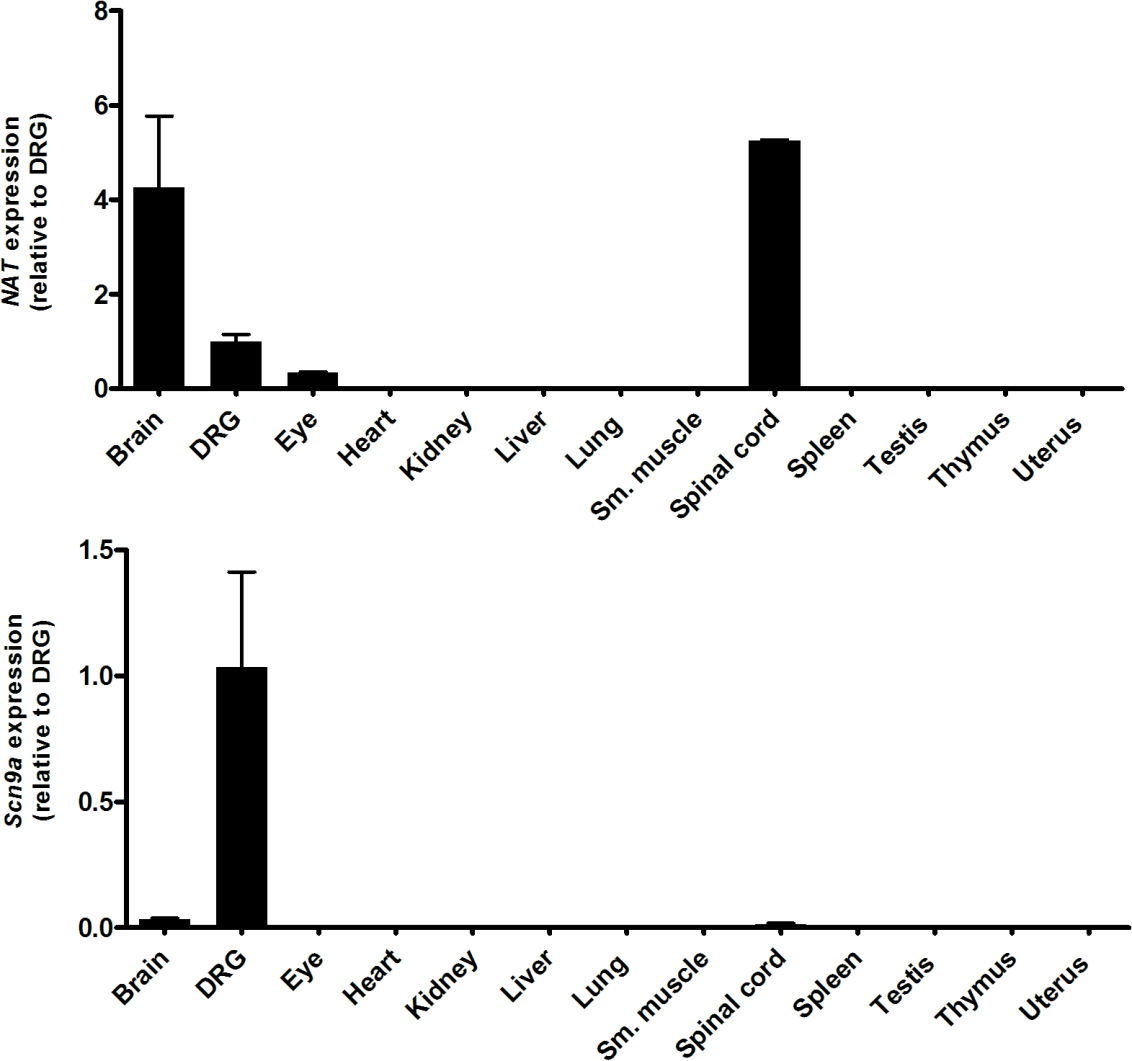
Real-time qPCR assays measuring the expression level of the NAT (A; upper panel) and *Scn9a* (B; lower panel) in specific mouse tissues (expression levels relative to DRG). Both the sense and NAT genes are expressed in similar tissues.

To investigate the tissue expression profile of the *Scn9a* NAT compared with the sense gene, we ran qPCR assays across a range of mouse tissue cDNA samples (Fig 2). The *Scn9a* sense and NAT genes have a relatively restricted expression pattern and are co-expressed in adult brain, DRG and spinal cord tissues. In addition, the NAT also shows expression within adult eye. The co-expression of the sense and NATs in similar tissues suggests that the NAT could have a direct regulatory effect on *Scn9a* gene functions. To further understand the relative expression of the NAT and *Scn9a* within DRG neurons, we analysed data from a recent paper in which DRG neurons have been categorized into 11 subtypes based on single-cell RNA-seq expression data (S3 Fig) [16]. This shows that the NAT is expressed in six of the eleven DRG neuronal subtypes. Interestingly, in the remaining five DRG subtypes with no NAT expression detected, there is robust *Scn9a* expression. Conversely, in the only neuronal subtype without *Scn9a* expression (‘NF5’), there is relatively high expression of the NAT, indicating that within particular neuronal cell populations there are contrasting expression profiles of the NAT and *Scn9a*.

### Overexpression of the human NAT specifically reduces Na_v_1.7 peak sodium currents

**Fig 3:**
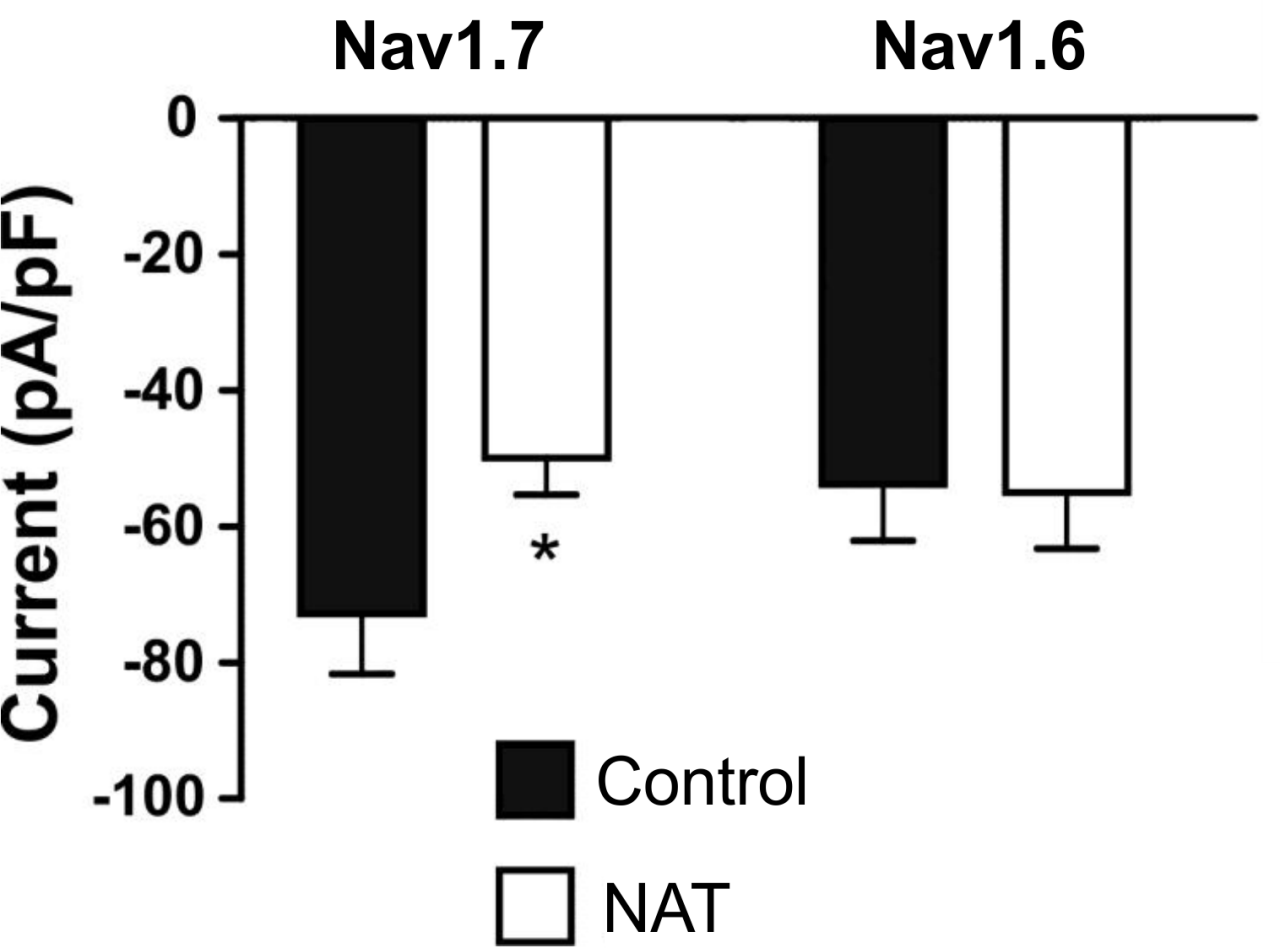
Overexpression of the human NAT inhibits Na_v_1.7 but not Na_v_1.6 currents. Whole cell voltage clamp recording from HEK cells stably expressing human Na_**v**_1.7 or human Na_**v**_1.6. Cells were transiently transfected with either pcDNA3 vector (black bars) or Na_**v**_1.7 NAT (white bars) along with a GFP-expressing vector and recorded from 48 hrs later. Peak whole cell current (pA) was measured in response to a 10 ms voltage step from the holding potential of -120 mV to 0 mV and normalized to cell size (pF). n = 17–20; * indicates p<0.05 when compared to control (unpaired t-test).

To functionally test the effects of overexpressing the human NAT on sodium currents, HEK293 cells stably expressing either Na_v_1.7 or Na_v_1.6 were transfected with (1) NAT in pcDNA3 plus a GFP-expressing vector (pEGFP-N1) or (2) empty pcDNA3 plus pEGFP-N1. Two days after transfection the green fluorescing cells were patch clamped. Overexpression of the human *SCN9A* NAT in the Na_v_1.7 stable cell line resulted in a statistically significant reduction in the peak sodium current (Fig 3). In Na_v_1.6 stably expressing cells, overexpression of the NAT had no effect on the sodium current, indicating that the NAT specifically affects the activity of Na_v_1.7.

### Stable expression of the human NAT in SH-SY5Y neuroblastoma cells

**Fig 4:**
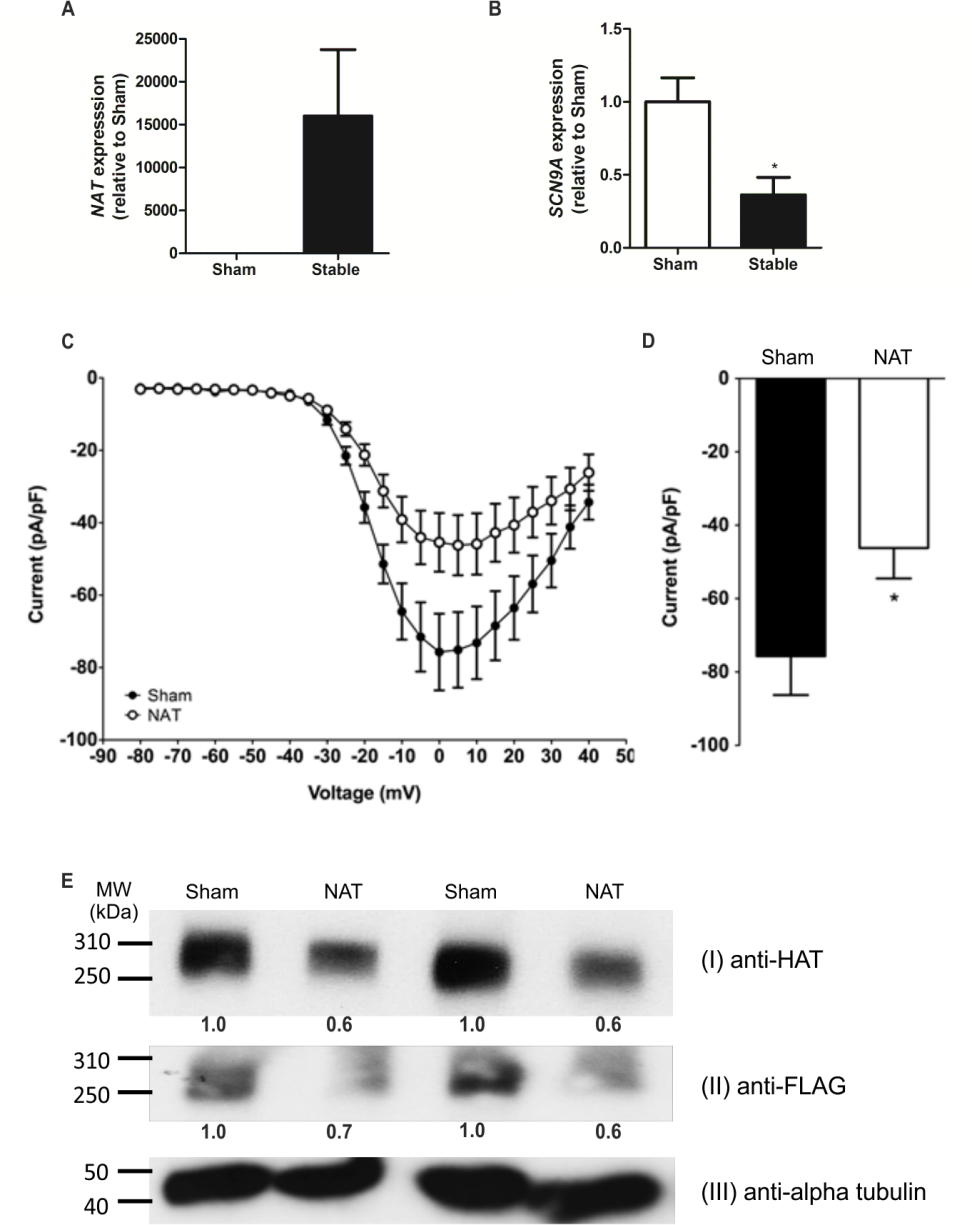
Overexpression of the NAT reduces Na_v_1.7-mRNA, protein and currents. (A) Real-time qPCR showing that the NAT is not expressed in naïve SH-SY5Y cells (sham) but is expressed in the stable-*NAT* SH-SY5Y cell line (n = 3). (B) Real-time qPCR showing that the endogenous *SCN9A* mRNA level is significantly reduced in the stable-NAT SH-SY5Y cell line compared to in naïve SH-SY5Y cells (sham) (n = 3). (C-D) Whole cell voltage clamp recordings of naïve (sham) and stable-NAT SH-SYSY (Na_**v**_1.7 NAT) cells showed a significant reduction of the peak sodium current (D), with voltage-current relationships unaltered (C) (n = 16–21). (E) Reduction in Na_**v**_1.7-TAP tag protein levels due to overexpression of the NAT for 48 hrs in a Na_**v**_1.7-TAP stable HEK293 cell line. Densitometry readings comparing alpha-tubulin to either HAT or FLAG bands were normalized to the sham controls and shown below each band. Transfection of the NAT results in a reduction in Na_**v**_1.7 protein. (Ei) Na_**v**_1.7-TAP protein detected by immunoblotting using an anti-HAT antibody following pull-down of Na_**v**_1.7-TAP with an anti-FLAG antibody (sham corresponds to mock-transfected cells). (Eii) Immunoblot of crude lysate using an anti-FLAG antibody confirms a reduction in Na_**v**_1.7 protein levels following NAT transfection. (Eiii) Immunoblot of crude lysate using an antibody to a house-keeping protein, confirming equal loading. * indicates p<0.05 when compared to control (unpaired t-test).

The SH-SY5Y neuroblastoma cell line endogenously expresses the *SCN9A* gene [17] but does not co-express the NAT (Fig 4A). We generated a SH-SY5Y cell line that stably expresses the human NAT cDNA under the control of a CMV promoter to assess the effect of overexpression of the NAT on endogenously-expressed Na_v_1.7. The *SCN9A* mRNA level in the NAT-stable cell line was significantly downregulated compared to wild-type SH-SY5Y cells (Fig 4B). Furthermore, patch clamping of this cell line showed a statistically significant reduction in the peak sodium current compared to a SH-SY5Y cell line that did not express the human NAT (Fig 4C and 4D). Voltage-current relationships were unaltered between the two cell lines.

In addition to sharing overlapping sequences with *SCN9A*, the NAT also contains sequences overlapping with two exons from each of *SCN1A*, *SCN2A* and *SCN3A* (S4 Fig). As the NAT shows high expression in the brain it is possible that the NAT may also regulate the Na_v_1.1-Na_v_1.3 brain-expressed sodium channels encoded by *SCN1A*-*SCN3A*. We were therefore interested to determine whether overexpression of the NAT downregulated endogenously expressed Na_v_1.1, Na_v_1.2 or Na_v_1.3 in the NAT-stable SH-SY5Y cell line. Real-time qPCR showed the expression level of *SCN2A* and *SCN3A* were not significantly different between the naïve SH-SY5Y cells (sham) and the stable-*NAT* SH-SY5Y cells (*SCN1A* expression could not be detected in the SH-SY5Y cell lines) (S5 Fig), further indicating that the functional effects of the NAT are *SCN9A*-specific.

Next, the effect of NAT overexpression on Na_v_1.7 protein levels was investigated. A HEK293 cell line was generated which stably expressed an epitope-tagged (TAP) Na_v_1.7 under the control of a CMV promoter. This cell line was transiently transfected with the human NAT, and anti-FLAG coupled Dynabeads were used to pull down TAP-tagged Na_v_1.7, which was subsequently detected on a western blot using an anti-HAT antibody. Comparison of NAT transfected and sham transfected cells showed that the transient NAT transfection resulted in a reduction in TAP-tagged Na_v_1.7 protein levels (Fig 4E). This reduction in protein level was further confirmed by immunoblotting using a pan sodium channel antibody (S6 Fig).

### *NAT* and *Scn9a* mRNA levels are unchanged in inflammatory and neuropathic pain states

**Fig 5:**
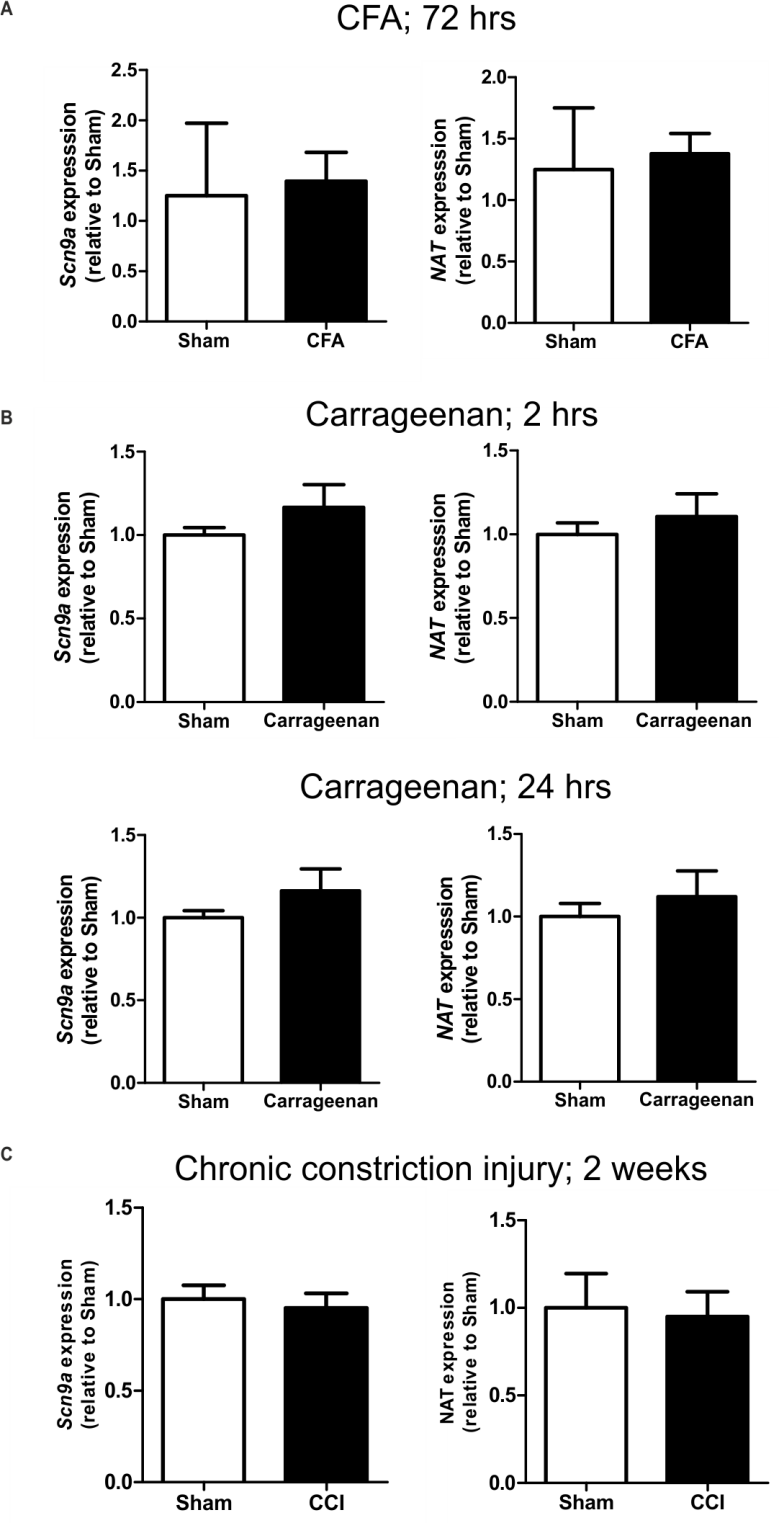
*Scn9a* and NAT expression levels in DRG do not change significantly in inflammatory and neuropathic pain models. Real-time qPCR assays for (A) CFA-induced pain (saline n = 3, CFA n = 4); (B) carrageenan-induced pain (saline n = 4, carrageenan n = 4); (C) chronic constriction injury (mock n = 6, CCI n = 6). Sham refers to saline injection (A, B) or mice undergoing a mock surgery (C).

Given that the NAT specifically downregulates human Na_v_1.7 protein levels and attenuates its currents, we investigated whether the NAT has a role *in vivo* to regulate mouse *Scn9a* mRNA levels in different pain states. Two different inflammatory pain mouse models (carrageenan and complete Freund’s adjuvant (CFA)) and one neuropathic pain model (chronic constriction injury) were used. Injection of carrageenan into the hind paw induces an acute and highly reproducible inflammatory response resulting in oedema, hyperalgesia and erythema that can persist for 6 days [18]. Likewise, injection of CFA results in acute thermal and mechanical hyperalgesia that can persist for more than 2 weeks. In the chronic constriction injury model, sutures are tied around the sciatic nerve which leads to thermal and mechanical hyperalgesia presenting within the first week and persisting for several weeks. Real-time qPCR assays using RNA isolated from ipsilateral L4-L6 dorsal root ganglia dissected 3 days (CFA), 2 hrs and 24 hrs (carrageenan) and 2 weeks (CCI) following the start of each pain model showed that neither *Scn9a* sense nor NAT mRNA levels change significantly (Fig 5A–5C).

## Discussion

We have identified, cloned and characterized a human and mouse natural antisense transcript for the *SCN9A* gene. Both human and mouse NATs are organised in a tail-to-tail configuration with the sense gene and both show evidence of alternative splicing, which is consistent with other reported NATs [1]. Although the mouse *Scn9a* NAT has fewer exons than the human NAT, both contain conserved sequences that overlap the final sense *SCN9A*/*Scn9a* exon. Given this conserved gene structure and the similar tissue expression profile of the sense and NAT genes, we hypothesized that the NAT regulates the function of *SCN9A*. We overexpressed the NAT in human cell lines that endogenously expressed Na_v_1.7 and in cells that stably expressed from a CMV promoter either Na_v_1.7 or epitope-tagged Na_v_1.7. Real-time qPCR, immunoblotting and electrophysiological assays showed a significant reduction in Na_v_1.7 mRNA, protein levels and currents.

How do NATs regulate the function of their sense gene counterparts? Characterization of other NATs has ascribed to them diverse cellular functions including transcriptional collision, RNA interference, regulation of alternative splicing, RNA editing, epigenetic regulation, RNA masking, translation inhibition and mRNA destabilisation [19]. In our experimental set-up we overexpressed the processed NAT from a CMV promoter in cell lines. This led to a reduction in sense mRNA and consequently a reduction in functional Na_v_1.7 protein. The NAT might function by downregulating *SCN9A* transcription, for example by guiding chromatin-modifying enzymes to the *SCN9A* promoter region. Alternatively, overexpression of the NAT could be leading to the generation of endogenous short RNAs (endo-siRNAs) thus leading to promotion of the RNA interference pathway [20]. A third mechanism could be that the NAT:sense mRNA duplexes are promoting destabilisation and degradation of the sense mRNA and/or inhibiting translation. A greater insight into the function of the NAT *in vivo* would be gleaned from the creation of a NAT knockout mouse. In a comparable experiment, Komine et al. generated a *Zfhx2-AS* knockout mouse through deletion of the transcriptional start site region for its corresponding NAT [21]. Normally, the *Zfhx2* expression pattern in the developing brain is complementary to the expression of its NAT. However in the *Zfhx2-AS* knockout mouse, the expression of the Zfhx2 homeobox-containing transcription factor becomes dysregulated. It would be interesting to determine whether the expression pattern of *Scn9a* is altered in a *Scn9a* NAT knockout mouse. Furthermore, the effects on transcription of other genes, which may also be potentially regulated by the NAT, could also be investigated in a NAT knockout, for example *Scn1a* and *Scn7a*, which reside in the same chromosomal region as *Scn9a*.

Detailed analyses of *Scn9a* knockout mice have shown that acute, inflammatory and some forms of neuropathic pain require the expression of Na_v_1.7 [22–24]. We hypothesized that the NAT may be downregulated in pain states, hence leading to upregulation of *SCN9A*. To test this, we assessed mRNA levels of the sense and NAT genes in CFA and carrageenan-induced models of inflammatory pain and in the chronic constriction injury neuropathic pain model. This showed that the mRNA level of neither the sense nor the NAT gene was significantly altered. The lack of change in the sense gene mRNA was a surprising finding given that, for example, Na_v_1.7 protein levels have previously been shown to increase following carrageenan injections [25]. Further work on epitope-tagged Na_v_1.7 knockin mice using anti-FLAG/HAT antibodies should help to confirm whether protein levels change in different pain models. Furthermore, intrathecal NAT overexpression by viral delivery could help to determine whether the NAT is able to confer analgesia and reduce the pain experienced in different inflammatory and neuropathic pain states.

In summary, this DRG-expressed natural antisense transcript attenuates native sodium currents, is co-expressed with its corresponding gene, and consequently has the potential to regulate pain thresholds via transcriptional and post-transcriptional regulation of *SCN9A*. Given that the presence of the NAT reduces Na_v_1.7 currents, then intuitively the lack of the NAT may increase sodium currents. This could lead to increased excitability of damage-sensing neurons, enabling a fine-control of responses to painful stimuli. Other regulatory mechanisms mediated by the NAT are also plausible, as discussed above. It will be interesting to determine whether loss of function mutations in the NAT are responsible for inherited painful disorders that map to human chromosome 2, and whether SNPs within the NAT alter pain thresholds in the general population.

## Material and Methods

### Cloning the human and mouse NATs

Human dorsal root ganglia total RNA was purchased from Clontech; mouse total RNA was isolated from DRGs dissected from wild-type C57BL/6 mice using the RNeasy MinElute Kit (Qiagen). Total RNA was reverse transcribed into cDNA using Superscript III (Life Technologies) and oligo d(T), according to the manufacturer’s instructions. The human *SCN9A* NAT was PCR-amplified from DRG cDNA using KAPA HiFi DNA polymerase (Kapa Biosystems) or LA Taq (TaKaRa) in three overlapping fragments using the following primers: Fragment 1, 5’ GAA TGA AAT TTA GTG TTT CCC ATC C, 5’ AGC AAT GTT TCA CCT CCA GAG ATC; Fragment 2, 5’ GGA ATT CAG GCA AAG TTG GA, 5’ CAC CAA CAT TCA GCC ATT TG; Fragment 3, 5’ CTG ATT ATT GGG AGA CTT TTG GAG, 5’ GCT CCT ATT TCT GAG TTT ATA CTG TG. The mouse *Scn9a* NAT was PCR-amplified using the primers 5’ agc aag agt aag aag tat tgg c, 5’ cat tat att tca ttt taa tg. PCR products were cloned using the Zero Blunt TOPO PCR Cloning Kit (Life Technologies). Individual colonies were Sanger sequenced and submitted to GenBank under the accession numbers KM096550 (clone LA5), KM096551 (clone LA8), KM096552 (clone 3.2) and KM096553 (clone 3.1).

### Real-time qPCR assay for mouse tissue panel

One μg of total RNA derived from a range of tissues (Clontech) or prepared in-house from DRG was reverse-transcribed into cDNA using Superscript III (Life Technologies) and oligo d(T). Real-time qPCR using technical triplicates was performed using SYBR Green (Applied Biosystems) and the following primers: Mouse *Scn9a* 5’ GAG GGG CAA ACT GAC TAC A and 5’ AGA AAC ATT CCT ACA ATG GAG (efficiency 1.91); Mouse NAT 5’ TGC TGT CAA CTC CTG AAC CA and 5’ TCC AAC TTT GCC ACA ATG AG (efficiency 1.96); Mouse *Actb* 5’ TTC TTT GCA GCT CCT TCG TT and 5’ ATG GAG GGG AAT ACA GCC C (efficiency 1.83). Relative expression of the target gene was calculated using the comparative ∆∆CT method [26,27]. Briefly, expression of the test gene was compared with that of *Actb* measured on the same sample, giving a CT difference (∆CT) for *Actb* minus the test gene. The relative expression of *Scn9a* and the NAT in specific tissues was calculated in relation to the expression levels in DRG using the comparative ∆∆CT method [26]. ∆CT values of individual tissues were compared to ∆CT of DRG, giving ∆∆CT values of a specific tissue and relative expression was calculated as 2-^∆∆CT^. Mean, standard error and statistics were calculated from the ∆∆CT data. Data were analysed using Microsoft Excel, and statistics were calculated using GraphPad Prism 5.01 software (GraphPad Software, San Diego, CA).

### Cloning the human NAT into an expression vector

The human *SCN9A* NAT was PCR-amplified from IMAGE clone 5582960 (BC051759) using Phusion High-Fidelity DNA polymerase (NEB) and primers 5’ CCC AAG CTT gtc tta gtc ctc tga ata ttt t and 5’ CGG GGT ACC CCA ATT GAT GGA GAA TTT TAT. The PCR product was then cloned into the *Hind*III and *Kpn*I restriction sites of pcDNA3 (Life Technologies) and fully sequenced. The cloning was designed so that the NAT was inserted at the most 5’ end of the multiple cloning site and near to the transcription start site of the vector, to minimise the additional nucleotides that were added to the 5’ end of the transcribed long ncRNA.

### Transfection of cell lines

HEK293 cells stably expressing human Na_v_1.7 plus *SCN1B* (L11) or Na_v_1.6 plus *SCN1B* (L40) (Scottish Biomedical) were grown according to the manufacturer’s instructions and passaged four times before transfections began. Cells were split into 3.5 cm dishes and grown to 95% confluency. On the day of transfection, cells were transfected with 250 ng of pEGFP-N1 (Clontech) plus 1000 ng of human NAT in pcDNA3 or empty pcDNA3 using a ratio of 1 μg DNA:2.5 μl Lipofectamine 2000 (Life Technologies). After 6 hours, the transfected cells were re-seeded at a low density and incubated for 48 hours prior to patch clamp analysis. Two independent transfections were tested.

### NAT-SH-SY5Y stable cell line

The human neuroblastoma cell line, SH-SY5Y (Public Health England) was cultured at 37°C/5% CO_2_ in Ham’s F12:EMEM (1:1) supplemented with 2 mM glutamine, 1% non essential amino acids and 10% foetal bovine serum (Life Technologies). The human NAT in pcDNA3 was linearised with *Pvu*I and following gel purification (Qiagen), 10 ug was transfected into a 10 cm 80% confluent dish of SH-SY5Y cells using Lipofectamine 2000 (Life Technologies). Six hours later, the medium was replaced with the selection antibiotic G418 (500 μg/ml). Cells were monitored on a daily basis until twenty-four discrete colonies could be selected for expansion and screening by RT-PCR. Real-time qPCR was performed using SYBR Green (Applied Biosystems) and the following primers: Human *SCN9A* 5’ AGA GGG GTA CAC CTG TGT GAA and 5’ CCC AGG AAA ATC ACT ACG ACA AA (efficiency 1.9); Human NAT 5’ GGA GTC ACT GGG ATT AAA GGC AT and 5’ TTC TTT GTC GCT GGT GGC TAG AG (efficiency 2); Human *ACTB* 5’ CGC CGC CAG CTC ACC ATG and 5’ CAC GAT GGA GGG GAA GAC GG (efficiency 1.85). The expression levels of *SCN1A*, *SCN2A* and *SCN3A* in the SH-SY5Y cells was determined using Taqman assays (Life Technologies) according to the manufacturer’s instructions. The following probes were used *SCN1A* (Hs00374696_m1), *SCN2A* (Hs01109877_m1), *SCN3A* (Hs00366902_m1) and *GAPDH* (Hs02758991_g1). Relative expression levels of mRNA were calculated using the comparative ΔΔCt (Ct) method and statistical significance was determined using an unpaired t-test.

### Patch clamp analysis of Na_v_1.7-HEK293, Na_v_1.6-HEK293 and NAT-SH-SY5Y stable cell lines

Whole cell patch clamp recordings were made from Na_v_1.7-HEK293, Na_v_1.6-HEK293 and NAT-SH-SY5Y stable cell lines at room temperature. In the case of the transient Na_v_1.7 NAT transfections, whole-cell membrane current recordings were performed 46 to 78 hrs after transfection. Micropipettes were pulled from borosilicate glass capillaries (GC150F-10; Harvard Apparatus, Kent, UK) using a Brown-Flaming P-97 horizontal micropipette puller (Sutter Instruments, Novato, CA, USA) and then fire polished on a microforge (MF-830 Narishige Group, Tokyo, Japan). Voltage errors were minimised with correction and prediction mode of series resistance compensation both set to 50%. Extracellular solution contained (in mmol/L): 140 NaCl, 4 KCl, 2 CaCl_2_, 1 MgCl_2_, 10 HEPES, adjusted to pH 7.4 with NaOH, osmolarity 320–325 mOsm/L with glucose. Pipettes were filled with an intracellular solution containing (in mmol/L): 140 CsCl, 5 NaCl, 5 EGTA, 2 MgCl_2_, 10 HEPES adjusted to pH 7.3 with CsOH, osmolarity 305–310 mOsm/L with glucose. Once filled with the appropriate intracellular solution, recording electrodes had a resistance between 2.0 and 3.0 MΩ. A silver chloride coated silver wire served as a reference electrode with one end connected to the ground input of the amplifier and the tip placed directly into the bath solution. Cells having a leak current after establishing whole-cell configuration of more than 10% of the peak sodium current were discarded and those which had developed leak of this magnitude during the experiment were not used in the final analysis. The liquid junction potential between the bath and the pipette solutions was not corrected. Whole-cell membrane currents were filtered at 5 kHz and sampled at 20 kHz using either an Axopatch 200B patch clamp amplifier or Axon Multiclamp 700B (Molecular Devices, Foster City, CA) and Digidata 1200B A/D converter (Molecular Devices, Foster City, CA). Data were acquired on a Windows-based PC using Clampex (Molecular Devices, Foster City, CA) software and analysed by pCLAMP (Clampfit) 9.2 software (Molecular Devices, Foster City, CA).

To characterize the voltage dependency of steady-state channel activation, currents were evoked by voltage increments of 5 mV from -80 to +40 mV for 10 ms from a holding potential of -120 mV with 5 s between pulses. Peak whole cell currents (pA) were measured in response to a 10 ms voltage step from a holding potential of -120 mV to 0 mV and normalized to cell capacitance (pF).

### Na_v_1.7-TAP tag stable HEK293A cell line

The human *SCN9A* mammalian expression construct, FLB [8] was modified by cloning in a sequence encoding a TAP tag (peptide: SRK DHL IHN VHK EEH AHA HNK IEN LYF QGE LPT AAD YKD HDG DYK DHD IDY KDD DDK) immediately prior to the stop codon. The TAP tag at the extreme C-terminus of Na_v_1.7 is comprised of a HAT domain and 3 FLAG tags, enabling immunodetection with either anti-HAT or anti-FLAG antibodies. The Na_v_1.7-TAP stable HEK293A cell line was generated using the same methods as described for the NAT-SH-SY5Y stable cell line. For the immunoprecipitation experiments, the Na_v_1.7-TAP stable HEK293A cell line was firstly transiently transfected with the human NAT expression construct or an empty pcDNA3 control (as described above) and cells collected 48 hrs later. Protein was isolated using RIPA buffer and equal amounts loaded onto 100 ul anti-FLAG (Sigma, F1804) coupled Dynabeads (Life Technologies), according to the manufacturer’s instructions. Samples were then boiled in Laemmli buffer and equal volumes loaded onto 4–12% polyacrylamide gels. Immunopurified Na_v_1.7-TAP was detected on immunoblots using an anti-HAT antibody (LSBio, LS-C51508). In addition, equal amounts of crude lysate protein were subjected to immunoblotting and detected using anti-FLAG, anti-alpha tubulin (Abcam, ab7291), anti-pan sodium channel (SIGMA, S8809) and anti-beta actin (Santa Cruz) antibodies. Densitometry readings were performed using ImageJ software whereby the region of interest (ROI) reading for alpha-tubulin was compared to the ROI reading for the HAT or FLAG bands and subsequently normalized to the sham control.

### Murine pain behaviour models

All experiments were performed in accordance with the UK Animals (Scientific Procedures) Act 1986 with prior approval under a Home Office project license (PPL 70/7382). Two different inflammatory pain models were used: intraplantar injection into the left hind paw of carrageenan or complete Freund’s adjuvant (or 0.9% sodium chloride solution for the sham controls), as described in [18]. The chronic constriction injury (CCI) model of neuropathic pain was also used, in which sutures were tied around the sciatic nerve [18]. All experiments were performed using 6–8 week old male C57BL/6 mice. Total RNA was isolated from dissected L4-L6 ipsilateral DRGs using the RNeasy MinElute Kit (Qiagen). Real-time qPCR was performed using SYBR Green (Applied Biosystems) and the following primers: Mouse *Scn9a* 5’ GAG GGG CAA ACT GAC TAC A and 5’ AGA AAC ATT CCT ACA ATG GAG (efficiency 1.91); Mouse NAT 5’ TGC TGT CAA CTC CTG AAC CA and 5’ TCC AAC TTT GCC ACA ATG AG (efficiency 1.96); Mouse *Gapdh* 5’ TGC GAC TTC AAC AGC AAC TC and 5’ CTT GCT CAG TGT CCT TGC TG (efficiency 1.76); Mouse *Actb* 5’ TTC TTT GCA GCT CCT TCG TT and 5’ ATG GAG GGG AAT ACA GCC C (efficiency 1.83). Relative expression levels of mRNA were calculated using the comparative ΔΔCt (Ct) method and statistical significance was determined using an unpaired t-test.

## Supporting Information

S1 Fig Alignment of *SCN9A* coding exons (NM_002977) with complementary *SCN9A* NAT sequence (NR_110260). Na_v_1.7 protein sequence (NP_002968) and location of human mutations associated with pain disorders highlighted.
(DOCX)

S2 Fig Translation in three frames of the 2 human and 2 mouse NAT splice variants cloned from dorsal root ganglion cDNA. Potential open reading frames beginning with a methionine residue are highlighted in red. Stop codons are denoted by a hyphen.
(DOCX)

S3 Fig Expression profile (fraction of positive cells by thresholding method) for *Scn9a* and the NAT (*Gm13629*) across 11 genetically defined DRG neuronal subtypes. The population size and the fraction of the population that would correspond to one cell are shown at the top. Data taken from Usoskin et al., 2015.
(DOCX)

S4 Fig Alignment of *SCN1A* exons (NM_001165963), *SCN2A* exons (NM_021007) and *SCN3A* exons (NM_006922) with complementary *SCN9A* NAT sequence (NR_110260). For *SCN4A* (NM_000334), *SCN5A* (NM_198056), *SCN7A* (NM_002976), *SCN8A* (NM_014191), *SCN10A* (NM_006514) and *SCN11A* (NM_014139) there was no significant similarity found with the *SCN9A* NAT (NR_110260).
(DOCX)

S5 Fig Real-time qPCR showing that the endogenous *SCN2A* (A) and *SCN3A* (B) mRNA levels are not significantly different in the stable-NAT SH-SY5Y cell line compared to in naïve SH-SY5Y cells (sham) (n = 3). *SCN1A* is not expressed in these cell lines.
(DOCX)

S6 Fig Reduction in Na_v_1.7-TAP tag protein levels due to overexpression of the NAT for 48 hrs in a Na_v_1.7-TAP stable HEK293 cell line. Upper: Immunoblot of crude lysate using an anti-pan sodium channel antibody confirms a reduction in Na_v_1.7 protein levels following NAT transfection. Lower: Immunoblot of crude lysate using an antibody to the beta actin housekeeping protein, confirming equal loading.
(DOCX)
