# Supplementary material for: Regulation of Nav1.7: A Conserved SCN9A Natural Antisense Transcript Expressed in Dorsal Root Ganglia
Source: PLoS One. 2015 Jun 2;10(6):e0128830. doi: 10.1371/journal.pone.0128830 (PMC4452699; doi:10.1371/journal.pone.0128830)
Supplement: S1 Fig — Nav1.7 protein sequence (NP_002968) and location of human mutations associated with pain disorders highlighted. (DOCX) [file pone.0128830.s001.docx]

**S1 Fig.**

*SCN9A* ex7 AATATTTTTATTACTTGGAAGGATCCAAAGATGCTCTCCTTTGTGGTTTCAGCACAGATT

||||||||||||||||||||||||||||||||||||||||||||||||||||||||||||

NAT AATATTTTTATTACTTGGAAGGATCCAAAGATGCTCTCCTTTGTGGTTTCAGCACAGATT

*SCN9A* ex7 CAGG

||||

NAT CAGG

Na_v_1.7

302 YFYYLEGSKDALLCGFSTDS 321

*SCN9A* ex15 CTTAGTGTTGGCCATCATCGTCTTCATTTTTGCTGTGGTCGGCATGCAGCTCTTTGGTAA

||||||||||||||||||||||||||||||||||||||||||||||||||||||||||||

NAT CTTAGTGTTGGCCATCATCGTCTTCATTTTTGCTGTGGTCGGCATGCAGCTCTTTGGTAA

*SCN9A* ex15 GAGCTACAAAGAATGTGTCTGCAAGATCAATGATGACTGTACGCTCCCACGGTGGCACAT

||||||||||||||||||||||||||||||||||||||||||||||||||||||||||||

NAT GAGCTACAAAGAATGTGTCTGCAAGATCAATGATGACTGTACGCTCCCACGGTGGCACAT

*SCN9A* ex15 GAACGACTTCTTCCACTCCTTCCTGATTGTGTTCCGCGTG

||||||||||||||||||||||||||||||||||||||||

NAT GAACGACTTCTTCCACTCCTTCCTGATTGTGTTCCGCGTG

Na_v_1.7

860 LVLAIIVFIFAVVGMQLFGKSYKECVCKINDDCTLPRWHMNDFFHSFLIVFRV 912

A863P (erythromelalgia)[[1](#_ENREF_1)]

V872G (erythromelalgia)[[2](#_ENREF_2)]

Q875E (erythromelalgia)[[3](#_ENREF_3),[4](#_ENREF_4)]

R896Q (congenital insensitivity to pain)[[5](#_ENREF_5)]

W897X (congenital insensitivity to pain)[[6](#_ENREF_6)]

*SCN9A* ex17 AGATTAAACCGGTCAAGCTCCTCAGAGTGCAGCACAGTTGATAACCCTTTGCCTGGAGAA

||||||||||||||||||||||||||||||||||||||||||||||||||||||||||||

NAT AGATTAAACCGGTCAAGCTCCTCAGAGTGCAGCACAGTTGATAACCCTTTGCCTGGAGAA

*SCN9A* ex17 GGAGAAGAAGCAGAGGCTGAACCTATGAATTCCGATGAGCCAGAGGC

|||||||||||||||||||||||||||||||||||||||||||||||

NAT GGAGAAGAAGCAGAGGCTGAACCTATGAATTCCGATGAGCCAGAGGC

Na_v_1.7

1107 RLNRSSSSECSTVDNPLPGEGEEAEAEPMNSDEPE 1141

*SCN9A* ex26 CCTGTTCCGAGTGATCCGTCTTGCCAGGATTGGCCGAATCCTACGTCTAGTCAAAGGAGC

||||||||||||||||||||||||||||||||||||||||||||||||||||||||||||

NAT CCTGTTCCGAGTGATCCGTCTTGCCAGGATTGGCCGAATCCTACGTCTAGTCAAAGGAGC

*SCN9A* ex26 AAAGGGGATCCGCACGCTGCTCTTTGCTTTGATGATGTCCCTTCCTGCGTTGTTTAACAT

||||||||||||||||||||||||||||||||||||||||||||||||||||||||||||

NAT AAAGGGGATCCGCACGCTGCTCTTTGCTTTGATGATGTCCCTTCCTGCGTTGTTTAACAT

*SCN9A* ex26 CGGCCTCCTGCTCTTCCTGGTCATGTTCATCTACGCCATCTTTGGAATGTCCAACTTTGC

||||||||||||||||||||||||||||||||||||||||||||||||||||||||||||

NAT CGGCCTCCTGCTCTTCCTGGTCATGTTCATCTACGCCATCTTTGGAATGTCCAACTTTGC

Na_v_1.7

1597 LFRVIRLARIGRILRLVKGAKGIRTLLFALMMSLPALFNIGLLLFLVMFIYAIFGMSNF 1655

R1599X (congenital insensitivity to pain)[[7](#_ENREF_7)]

L1612P (cold sensitive paroxysmal extreme pain disorder (PEPD))[[8](#_ENREF_8)]

M1627K (PEPD)[[9](#_ENREF_9)]

A1632E (overlapping phenotype of erythromelalgia and PEPD) [[10](#_ENREF_10)]

A1632T (erythromelalgia)[[11](#_ENREF_11)]

1. Harty TP, Dib-Hajj SD, Tyrrell L, Blackman R, Hisama FM, Rose JB, et al. (2006) Na(V)1.7 mutant A863P in erythromelalgia: effects of altered activation and steady-state inactivation on excitability of nociceptive dorsal root ganglion neurons. J Neurosci 26: 12566-12575.

2. Choi JS, Zhang L, Dib-Hajj SD, Han C, Tyrrell L, Lin Z, et al. (2009) Mexiletine-responsive erythromelalgia due to a new Na(v)1.7 mutation showing use-dependent current fall-off. Exp Neurol 216: 383-389.

3. Stadler T, O'Reilly AO, Lampert A (2015) Erythromelalgia Mutation Q875E Stabilizes the Activated State of Sodium Channel Nav1.7. J Biol Chem 290: 6316-6325.

4. Skeik N, Rooke TW, Davis MD, Davis DM, Kalsi H, Kurth I, et al. (2012) Severe case and literature review of primary erythromelalgia: novel SCN9A gene mutation. Vasc Med 17: 44-49.

5. Cox JJ, Sheynin J, Shorer Z, Reimann F, Nicholas AK, Zubovic L, et al. (2010) Congenital insensitivity to pain: novel SCN9A missense and in-frame deletion mutations. Hum Mutat 31: E1670-1686.

6. Cox JJ, Reimann F, Nicholas AK, Thornton G, Roberts E, Springell K, et al. (2006) An SCN9A channelopathy causes congenital inability to experience pain. Nature 444: 894-898.

7. Mansouri M, Chafai Elalaoui S, Ouled Amar Bencheikh B, El Alloussi M, Dion PA, Sefiani A, et al. (2014) A novel nonsense mutation in SCN9A in a Moroccan child with congenital insensitivity to pain. Pediatr Neurol 51: 741-744.

8. Suter MR, Bhuiyan ZA, Laedermann CJ, Kuntzer T, Schaller M, Stauffacher MW, et al. (2015) p.L1612P, a novel voltage-gated sodium channel Nav1.7 mutation inducing a cold sensitive paroxysmal extreme pain disorder. Anesthesiology 122: 414-423.

9. Dib-Hajj SD, Estacion M, Jarecki BW, Tyrrell L, Fischer TZ, Lawden M, et al. (2008) Paroxysmal extreme pain disorder M1627K mutation in human Nav1.7 renders DRG neurons hyperexcitable. Mol Pain 4: 37.

10. Estacion M, Dib-Hajj SD, Benke PJ, Te Morsche RH, Eastman EM, Macala LJ, et al. (2008) NaV1.7 gain-of-function mutations as a continuum: A1632E displays physiological changes associated with erythromelalgia and paroxysmal extreme pain disorder mutations and produces symptoms of both disorders. J Neurosci 28: 11079-11088.

11. Eberhardt M, Nakajima J, Klinger AB, Neacsu C, Huhne K, O'Reilly AO, et al. (2014) Inherited pain: sodium channel Nav1.7 A1632T mutation causes erythromelalgia due to a shift of fast inactivation. J Biol Chem 289: 1971-1980.
