# Supplementary material for: Regulation of Nav1.7: A Conserved SCN9A Natural Antisense Transcript Expressed in Dorsal Root Ganglia
Source: PLoS One. 2015 Jun 2;10(6):e0128830. doi: 10.1371/journal.pone.0128830 (PMC4452699; doi:10.1371/journal.pone.0128830)
Supplement: S2 Fig — Potential open reading frames beginning with a methionine residue are highlighted in red. Stop codons are denoted by a hyphen. (DOCX) [file pone.0128830.s002.docx]

**S2 Fig.**

**Translation of KM096550 (human NAT splice variant 1):**

Frame 1:

PRDTQHPQLHTLMPLCIYARNTITKELDIIRQ-IKRY-QLRLSDGPCLGFGIFA-NRDSS

HQRQRSQGDCGL-EALPGNRNSGKVGHSKDGVDEHDQEEQEADVKQRRKGHHQSKEQRAD

PLCSFD-T-DSANPGKTDHSEQGNRDLWR-NIALNWH-F-PSSCRPITNLSISLQCLELD

LAHRQRVINCAAL-GA-PV-SLERNSPPHQHAEHNQEGVEEVVHVPPWERTVIIDLADTF

FVALTKELHADHSKNEDDDGQH-AFRLKRKQILHTLNLC-NHKGEHLWILPSNKNILLKK

NLNSYNITDFNL-L--RRSN-KI-LLGDFWSNHGRKTMKSDILGFECCFYPFLT-IHGHS

LSSVPNLAEPQSTHNLASVFSMLATKWLNVGGETLTTK-MVIF-FRSCCPGWSAMARSRL

TATSASRVQAILLPQPPE-LELQAPTTMPG-FFVFL

Frame 2:

RETPSIHNFTL-CHCVFMQEIP-LRNLTSSDSKSRGTDN-D-VMVPA-DLESLHEIGTLA

TSDKEVRETAVSRKHFQETGIQAKLDIPKMA-MNMTRKSRRPMLNNAGRDIIKAKSSVRI

PFAPLTRRRIRPILARRITRNRVTEISGGETLL-TGIDSSPALVDQLPTCPSPSSAWNWT

WHTGKGLSTVLHSEELDRFNL-KGIHHPTSTRNTIRKEWKKSFMCHRGSVQSSLILQTHS

L-LLPKSCMPTTAKMKTMMANTKPLD-KENKYYIP-ICAETTKESIFGSFQVIKIFC-RR

I-TVITSQTLICDCDKGGQIKKSDYWETFGVIMEERL-NQIFLDLNVASIPS-LKSMGIH

YHQCLTWQNHSLHIIWHLSFPCWLPNG-MLVEKLSQPSEWLYFNFALVAQAGVQWHDLGS

PQPLPPEFKRFSCLSLLSSWNYRRPPPCPANFLYF

Frame 3:

ERHPASTTSHSDAIVYLCKKYHN-GT-HHQTVNQEVLTTETE-WSLPRIWNLCMK-GL-P

PATKKSGRLRSLGSTSRKQEFRQSWTFQRWRR-T-PGRAGGRC-TTQEGTSSKQRAACGS

PLLL-LDVGFGQSWQDGSLGTG-QRSLEVKHCSELALILAQLL-TNYQLVHLPPVPGTGP

GTQAKGYQLCCTLRSLTGLISRKEFTTPPARGTQSGRSGRSRSCATVGAYSHH-SCRHIL

CSSYQRAACRPQQK-RR-WPTLSL-TKKKTNITYPESVLKPQRRASLDPSK--KYSVEEE

FEQL-HHRL-SVIVIKEVKLKNLIIGRLLE-SWKKDYEIRYSWI-MLLLSLLNLNPWAFI

IISA-PGRTTVYT-SGICLFHAGYQMAECWWRNSHNQVNGYILISLLLPRLECNGTISAH

RNLCLPSSSDSPASAS-VAGITGAHHHARLIFCIF

**Translation of KM096551 (human NAT splice variant 2):**

Frame 1:

PRDTQHPQLHTLMPLCIYARNTITKELDIIRQ-IKRY-QLRLSDGPCLGFGIFA-NRDSS

HQRQRSQGDCGL-EALPGNRNSGKVGHSKDGVDEHDQEEQEADVKQRRKGHHQSKEQRAD

PLCSFD-T-DSANPGKTDHSEQGNRDLWR-NIALNWH-F-PSSCRPITNLSISLQCLELD

LAHRPLAHRNS-VQPLLLLLLQAKGYQLCCTLRSLTGLISRKEFTTPPAFRLKRKQILHT

LNLC-NHKGEHLWILPSNKNILLKKNLNSYNITDFNL-L--RRSN-KI-LLGDFWSNHGR

KTMKSDILGFECCFYPFLT-IHGHSLSSVPNLAEPQSTHNLASVFSMLATKWLNVGGETL

TTK-MVIF-FRSCCPGWSAMARSRLTATSASRVQAILLPQPPE-LELQAPTTMPG-FFVF

L

Frame 2:

RETPSIHNFTL-CHCVFMQEIP-LRNLTSSDSKSRGTDN-D-VMVPA-DLESLHEIGTLA

TSDKEVRETAVSRKHFQETGIQAKLDIPKMA-MNMTRKSRRPMLNNAGRDIIKAKSSVRI

PFAPLTRRRIRPILARRITRNRVTEISGGETLL-TGIDSSPALVDQLPTCPSPSSAWNWT

WHTGLWLIGIHRFSLCFFSFSRQRVINCAAL-GA-PV-SLERNSPPHQPLD-KENKYYIP

-ICAETTKESIFGSFQVIKIFC-RRI-TVITSQTLICDCDKGGQIKKSDYWETFGVIMEE

RL-NQIFLDLNVASIPS-LKSMGIHYHQCLTWQNHSLHIIWHLSFPCWLPNG-MLVEKLS

QPSEWLYFNFALVAQAGVQWHDLGSPQPLPPEFKRFSCLSLLSSWNYRRPPPCPANFLYF

Frame 3:

ERHPASTTSHSDAIVYLCKKYHN-GT-HHQTVNQEVLTTETE-WSLPRIWNLCMK-GL-P

PATKKSGRLRSLGSTSRKQEFRQSWTFQRWRR-T-PGRAGGRC-TTQEGTSSKQRAACGS

PLLL-LDVGFGQSWQDGSLGTG-QRSLEVKHCSELALILAQLL-TNYQLVHLPPVPGTGP

GTQASGSSEFIGSASASSPSPGKGLSTVLHSEELDRFNL-KGIHHPTSL-TKKKTNITYP

ESVLKPQRRASLDPSK--KYSVEEEFEQL-HHRL-SVIVIKEVKLKNLIIGRLLE-SWKK

DYEIRYSWI-MLLLSLLNLNPWAFIIISA-PGRTTVYT-SGICLFHAGYQMAECWWRNSH

NQVNGYILISLLLPRLECNGTISAHRNLCLPSSSDSPASAS-VAGITGAHHHARLIFCIF

**Translation of KM096552 (mouse NAT splice variant 1):**

Frame 1:

SSKQAGRLRSCMEIMNQVLLFVFETFWKESYE-PRNRSHFVGRKNDQKHES-ISWQVQGT

SLGVCSKPEGTTSPAVNS-TITSGVKEGAGEAL--MQRMLLRALVCL-LIVAKLDIPKMA

-MNMTRKSRRPMLNSAGRDIIRAKSSVRIPLAPLIRRRIRPILARRMTRNRMLLPVFTEK

LL-PVNDSE-RNAWLLETPRVTAACSAQEDVHILLQGSRNKAW--QEDSKSPGHRDGSYE

ALCCTKDTINVIAAP-QRSAVGSCNTERVSIHSFIYREGPCSHIFSAEL-ATDGLWEKQT

FSSALGPLLSSLG-TSSMLAWFCSSYAMATTARIKFIR-KDPRNITTTKKIM-VLPAARS

VYGEQKRDMIILVIQKFH-GFYCS-YIMNVQTRYFCIILL-KDK-R-YL-GKSVLNKLFN

IKDTALLYVH-GLIL

Frame 2:

AASRRAG-DLAWKS-TRSCFSFLKRFGRRVMNSPEIGLILWVGRMTRSMKAKSPGKCRGP

LLECAVNPRGRLLLLSTPEPSHLE-RKGLVKPCNKCKGCC-ELWSAFNSLWQSWTSQRWR

R-T-RGKAGGRC-TAQEGTSSEQRAACGSPWRL-SGVGFVQSWPGE-LGTGCYSQSSLRN

CSSQ-TTVNEEMPGCSRHRE-QLRAQLRRTFISFFKALGTKRGDDRKTVRAQDIGMGAMK

LCAVPRTQSM-SRHHNRGQQWVRAILSGSAFIHSYTERAHAATSSLLNYELPMDCGRNKH

SLQPWAHF-VH-VKLLRCWPGSVPRTPWLPQPGSSLSGKRIPEISPRQRKSCRFCQQHAV

SMGSKKET-LF--YKNFTKVFIAASI--MCKPDIFV-FCCRKINKDNIFEENQY-TNFLI

-KIQHCYMYIKA-F

Frame 3:

QQAGGQAEILHGNHEPGLAFRF-NVLEGEL-IAQK-VSFCG-EE-PEA-KLNLLASAGDL

SWSVQ-TRGDDFSCCQLLNHHIWSEGRGW-SLVINAKDAAESFGLPLTHCGKVGHPKDGV

DEHDEEKQEADVEQRRKGHHQSKEQRADPLGAFDQA-DSSNPGQANDSEQDATPSLH-ET

ALASERQ-MKKCLAARDTESDSCVLSSGGRSYPSSRL-EQSVVMTGRQ-EPRT-GWEL-S

SVLYQGHNQCNRGTITEVSSGFVQY-AGQHSFIHIQRGPMQPHLLC-IMSYRWIVGETNI

LFSLGPTSEFTRLNFFDVGLVLFLVRHGYHSQDQVYQVKGSQKYHHDKENHVGFASSTQC

LWGAKKRHDYFSNTKISLRFLLQLVYNECANQIFLYNFVVER-IKIISLRKISIKQTF-Y

KRYSIVICTLRLDS

**Translation of KM096553 (mouse NAT splice variant 2):**

Frame 1:

SSKQAGRLRSCMEIMNQVLLFVFETFWKESYE-PRNRSHFVGRKNDQKHES-ISWQVQGT

SLGVCSKPEGTTSPAVNS-TITSGVKEGAGEAL--MQRMLLRALVCL-LIVAKLDIPKMA

-MNMTRKSRRPMLNSAGRDIIRAKSSVRIPLAPLIRRRIRPILARRMTRNRMLLPVFTEK

LL-PVNDSE-RNAWLLETPRVTAACSAQEDVHILLQGSRNKAW--QEDSKSPGHRDGSYE

ALCCTKDTINVIAAP-QSFFDVGLVLFLVRHGYHSQDQVYQVKGSQKYHHDKENHVGFAS

STQCLWGAKKRHDYFSNTKISLRFLLQLVYNECANQIFLYNFVVER-IKIISLRKISIKQ

TF-YKRYSIVICTLRLDS

Frame 2:

AASRRAG-DLAWKS-TRSCFSFLKRFGRRVMNSPEIGLILWVGRMTRSMKAKSPGKCRGP

LLECAVNPRGRLLLLSTPEPSHLE-RKGLVKPCNKCKGCC-ELWSAFNSLWQSWTSQRWR

R-T-RGKAGGRC-TAQEGTSSEQRAACGSPWRL-SGVGFVQSWPGE-LGTGCYSQSSLRN

CSSQ-TTVNEEMPGCSRHRE-QLRAQLRRTFISFFKALGTKRGDDRKTVRAQDIGMGAMK

LCAVPRTQSM-SRHHNRASSMLAWFCSSYAMATTARIKFIR-KDPRNITTTKKIM-VLPA

ARSVYGEQKRDMIILVIQKFH-GFYCS-YIMNVQTRYFCIILL-KDK-R-YL-GKSVLNK

LFNIKDTALLYVH-GLIL

Frame 3:

QQAGGQAEILHGNHEPGLAFRF-NVLEGEL-IAQK-VSFCG-EE-PEA-KLNLLASAGDL

SWSVQ-TRGDDFSCCQLLNHHIWSEGRGW-SLVINAKDAAESFGLPLTHCGKVGHPKDGV

DEHDEEKQEADVEQRRKGHHQSKEQRADPLGAFDQA-DSSNPGQANDSEQDATPSLH-ET

ALASERQ-MKKCLAARDTESDSCVLSSGGRSYPSSRL-EQSVVMTGRQ-EPRT-GWEL-S

SVLYQGHNQCNRGTITELLRCWPGSVPRTPWLPQPGSSLSGKRIPEISPRQRKSCRFCQQ

HAVSMGSKKET-LF--YKNFTKVFIAASI--MCKPDIFV-FCCRKINKDNIFEENQY-TN

FLI-KIQHCYMYIKA-F
