# Supplementary material for: Regulation of Nav1.7: A Conserved SCN9A Natural Antisense Transcript Expressed in Dorsal Root Ganglia
Source: PLoS One. 2015 Jun 2;10(6):e0128830. doi: 10.1371/journal.pone.0128830 (PMC4452699; doi:10.1371/journal.pone.0128830)
Supplement: S3 Fig — The population size and the fraction of the population that would correspond to one cell are shown at the top. Data taken from Usoskin et al., 2015. (DOCX) [file pone.0128830.s003.docx]

**S3 Fig.**


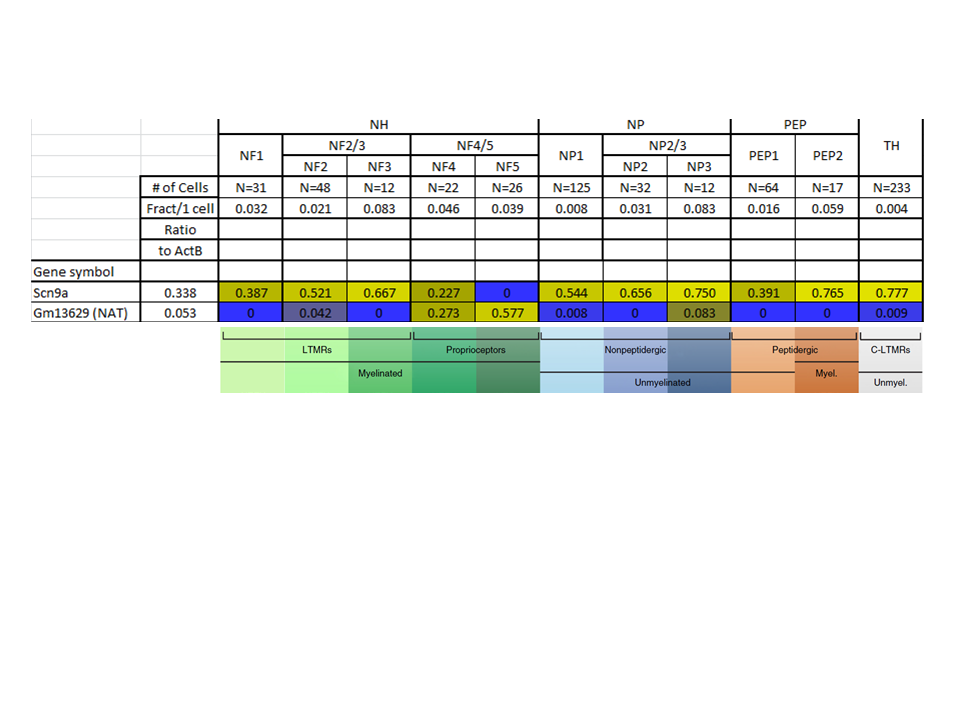


Usoskin D, Furlan A, Islam S, Abdo H, Lonnerberg P, Lou D, et al. (2015) Unbiased classification of sensory neuron types by large-scale single-cell RNA sequencing. Nat Neurosci 18: 145-153.

http://linnarssonlab.org/drg/
