# Supplementary material for: Regulation of Nav1.7: A Conserved SCN9A Natural Antisense Transcript Expressed in Dorsal Root Ganglia
Source: PLoS One. 2015 Jun 2;10(6):e0128830. doi: 10.1371/journal.pone.0128830 (PMC4452699; doi:10.1371/journal.pone.0128830)
Supplement: S4 Fig — For SCN4A (NM_000334), SCN5A (NM_198056), SCN7A (NM_002976), SCN8A (NM_014191), SCN10A (NM_006514) and SCN11A (NM_014139) there was no significant similarity found with the SCN9A NAT (NR_110260). (DOCX) [file pone.0128830.s004.docx]

**S4 Fig.**

Alignment of *SCN1A* exons (NM_001165963) with complementary *SCN9A* NAT sequence (NR_110260)

*SCN1A* ex15 TTGGCCATCATCGTCTTCATTTTTGCCGTGGTCGGCATGCAGCTCTTTGGTAAAAGCTAC

|||||||||||||||||||||||||| |||||||||||||||||||||||||| ||||||

NAT TTGGCCATCATCGTCTTCATTTTTGCTGTGGTCGGCATGCAGCTCTTTGGTAAGAGCTAC

*SCN1A* ex15 AAAGATTGTGTCTGCAAGATCGCCAG-TGATTGTCAACTCCCACGCTGGCACATGAATGA

||||| ||||||||||||||| | ||| ||| |||||||| ||||||||||| ||

NAT AAAGAATGTGTCTGCAAGATC-AATGATGACTGTACGCTCCCACGGTGGCACATGAACGA

*SCN1A* ex15 CTTCTTCCACTCCTTCCTGATTGTGTTCCGCGTGCTGTGTGGGG

||||||||||||||||||||||||||||||||||||| ||||||

NAT CTTCTTCCACTCCTTCCTGATTGTGTTCCGCGTGCTG-GTGGGG

*SCN1A* ex26 CTACCCTGTTCCGAGTGATCCGTCTTGCTAGGATTGGCCGAATCCTACGTCTGATCAAAG

|||||||||||||||||||||||||||| ||||||||||||||||||||||| ||||||

NAT CTACCCTGTTCCGAGTGATCCGTCTTGCCAGGATTGGCCGAATCCTACGTCTAGTCAAAG

*SCN1A* ex26 GAGCAAAGGGGATCCGCACGCTGCTCTTTGCTTTGATGATGTCCCTTCCTGCGTTGTTTA

||||||||||||||||||||||||||||||||||||||||||||||||||||||||||||

NAT GAGCAAAGGGGATCCGCACGCTGCTCTTTGCTTTGATGATGTCCCTTCCTGCGTTGTTTA

*SCN1A* ex26 ACATCGGCCTCCTACTCTTCCTAGTCATGTTCATCTACGCCATCTTTGGGATGTCCAACT

||||||||||||| |||||||| |||||||||||||||||||||||||| ||||||||||

NAT ACATCGGCCTCCTGCTCTTCCTGGTCATGTTCATCTACGCCATCTTTGGAATGTCCAACT

*SCN1A* ex26 TTGCCT

||||||

NAT TTGCCT

Alignment of *SCN2A* exons (NM_021007) with complementary *SCN9A* NAT sequence (NR_110260)

*SCN2A* ex15 CTTGGTATTGGCCATCATCGTCTTCATTTTTGCTGTGGTCGGCATGCAGCTCTTTGGTAA

||| || |||||||||||||||||||||||||||||||||||||||||||||||||||||

NAT CTTAGTGTTGGCCATCATCGTCTTCATTTTTGCTGTGGTCGGCATGCAGCTCTTTGGTAA

*SCN2A* ex15 GAGCTACAAAGAATGTGTCTGCAAGATTTCCAATGATTG-TGAA--CTCCCACGCTGGCA

||||||||||||||||||||||||||| | |||||| || | |||||||| |||||

NAT GAGCTACAAAGAATGTGTCTGCAAGAT--C-AATGATGACTGTACGCTCCCACGGTGGCA

*SCN2A* ex15 CATGCATGACTTTTTCCACTCCTTCCTGATCGTGTTCCGCGTGCTGTGTGG

|||| | ||||| ||||||||||||||||| ||||||||||||||| ||||

NAT CATGAACGACTTCTTCCACTCCTTCCTGATTGTGTTCCGCGTGCTG-GTGG

*SCN2A* ex26 CTACCCTGTTCCGAGTGATCCGTCTTGCCAGGATTGGCCGAATCCTACGTCTGATCAAAG

|||||||||||||||||||||||||||||||||||||||||||||||||||| ||||||

NAT CTACCCTGTTCCGAGTGATCCGTCTTGCCAGGATTGGCCGAATCCTACGTCTAGTCAAAG

*SCN2A* ex26 GAGCAAAGGGGATCCGCACGCTGCTCTTTGCTTTGATGATGTCCCTTCCTGCGTTGTTTA

||||||||||||||||||||||||||||||||||||||||||||||||||||||||||||

NAT GAGCAAAGGGGATCCGCACGCTGCTCTTTGCTTTGATGATGTCCCTTCCTGCGTTGTTTA

*SCN2A* ex26 ACATCGGCCTCCTTCTTTTCCTGGTCATGTTCATCTACGCCATCTTTGGGATGTCCAATT

||||||||||||| || |||||||||||||||||||||||||||||||| |||||||| |

NAT ACATCGGCCTCCTGCTCTTCCTGGTCATGTTCATCTACGCCATCTTTGGAATGTCCAACT

*SCN2A* ex26 TTGCCT

||||||

NAT TTGCCT

Alignment of *SCN3A* exons (NM_006922) with complementary *SCN9A* NAT sequence (NR_110260):

SCN3A ex15 CTTGGTGTTGGCCATCATCGTCTTCATTTTTGCTGTGGTCGGCATGCAGCTCTTTGGTAA

||| ||||||||||||||||||||||||||||||||||||||||||||||||||||||||

NAT CTTAGTGTTGGCCATCATCGTCTTCATTTTTGCTGTGGTCGGCATGCAGCTCTTTGGTAA

SCN3A ex15 GAGCTACAAAGAATGTGTCTGCAAGATCAATGATGACTGTACGCTCCCACGGTGGCACAT

||||||||||||||||||||||||||||||||||||||||||||||||||||||||||||

NAT GAGCTACAAAGAATGTGTCTGCAAGATCAATGATGACTGTACGCTCCCACGGTGGCACAT

SCN3A ex15 GAACGACTTCTTCCACTCCTTCCTGATTGTGTTCCGCGTGCTGTGTGG

||||||||||||||||||||||||||||||||||||||||||| ||||

NAT GAACGACTTCTTCCACTCCTTCCTGATTGTGTTCCGCGTGCTG-GTGG

SCN3A ex26 CTACCTTGTTCCGAGTGATCCGTCTTGCCAGGATTGGCCGAATCCTACGTCTGATCAAAG

||||| |||||||||||||||||||||||||||||||||||||||||||||| ||||||

NAT CTACCCTGTTCCGAGTGATCCGTCTTGCCAGGATTGGCCGAATCCTACGTCTAGTCAAAG

SCN3A ex26 GAGCAAAGGGGATCCGCACGCTGCTCTTTGCTTTGATGATGTCCCTTCCTGCGTTGTTTA

||||||||||||||||||||||||||||||||||||||||||||||||||||||||||||

NAT GAGCAAAGGGGATCCGCACGCTGCTCTTTGCTTTGATGATGTCCCTTCCTGCGTTGTTTA

SCN3A ex26 ACATCGGCCTCCTGCTCTTCCTGGTCATGTTTATCTATGCCATCTTTGGGATGTCCAACT

||||||||||||||||||||||||||||||| ||||| ||||||||||| ||||||||||

NAT ACATCGGCCTCCTGCTCTTCCTGGTCATGTTCATCTACGCCATCTTTGGAATGTCCAACT

SCN3A ex26 TTGCCT

||||||

NAT TTGCCT

For *SCN4A* (NM_000334), *SCN5A* (NM_198056), *SCN7A* (NM_002976), *SCN8A* (NM_014191), *SCN10A* (NM_006514) and *SCN11A* (NM_014139) there was no significant similarity found with the *SCN9A* NAT (NR_110260).
