# Supplementary figures and images for: Regulation of Nav1.7: A Conserved SCN9A Natural Antisense Transcript Expressed in Dorsal Root Ganglia
Source: PLoS One. 2015 Jun 2;10(6):e0128830. doi: 10.1371/journal.pone.0128830 (PMC4452699; doi:10.1371/journal.pone.0128830)

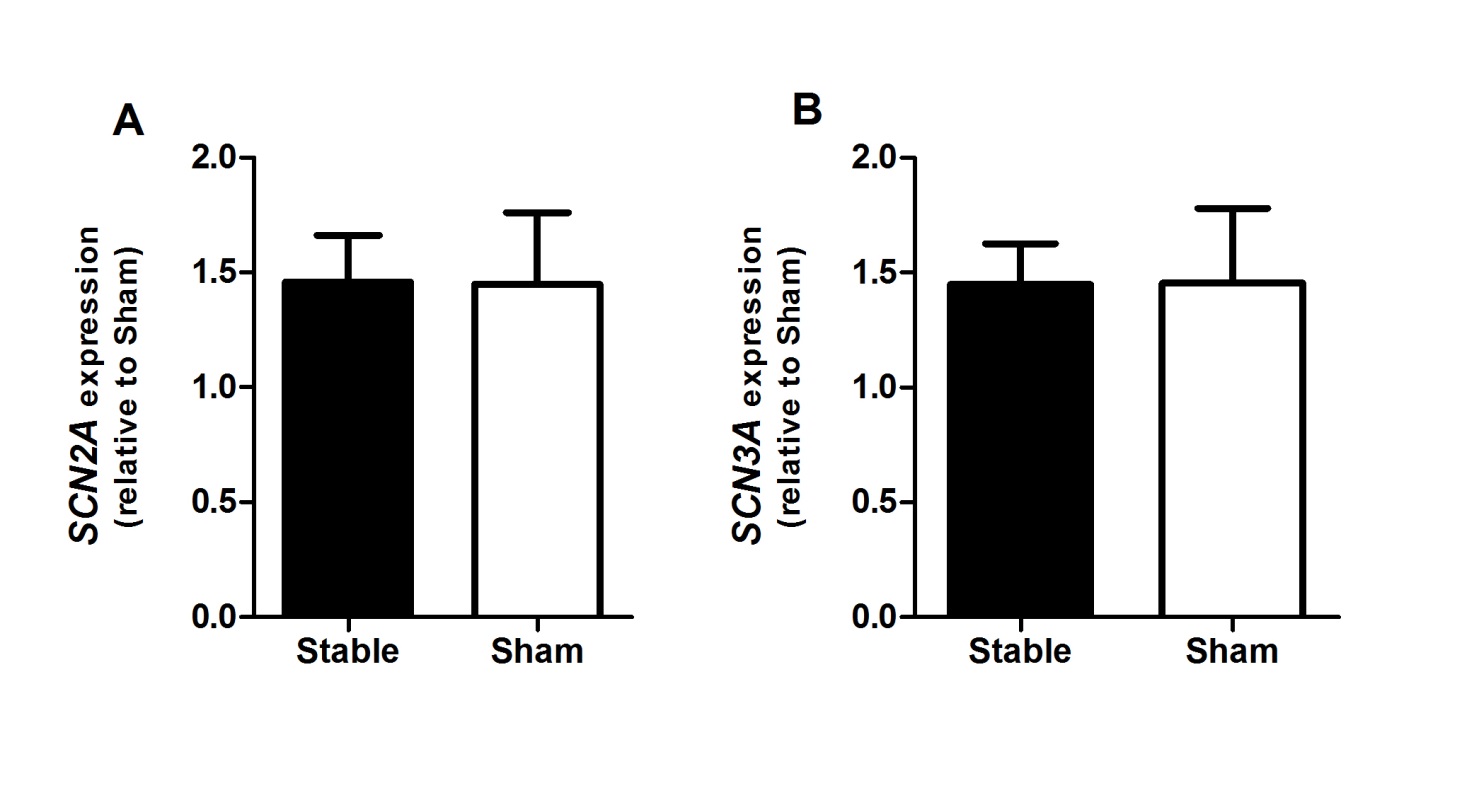
**S5 Fig.**

Supplement: S5 Fig — SCN1A is not expressed in these cell lines. (DOCX) [file pone.0128830.s005.docx]

**S6 Fig.**


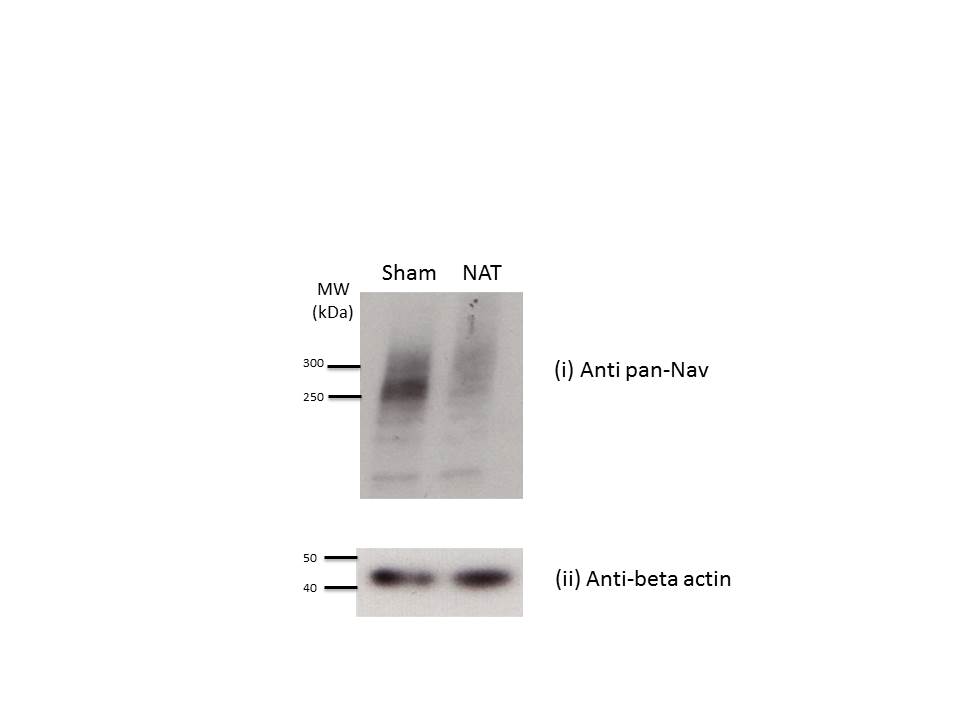

Supplement: S6 Fig — Upper: Immunoblot of crude lysate using an anti-pan sodium channel antibody confirms a reduction in Nav1.7 protein levels following NAT transfection. Lower: Immunoblot of crude lysate using an antibody to the beta actin housekeeping protein, confirming equal loading. (DOCX) [file pone.0128830.s006.docx]
